# Engaging adolescents in chronic disease prevention research: insights from researchers about establishing and facilitating a youth advisory group

**DOI:** 10.1186/s40900-024-00559-1

**Published:** 2024-02-26

**Authors:** Stephanie R. Partridge, Mariam Mandoh, Allyson Todd, Rebecca Raeside

**Affiliations:** 1 Susan Wakil School of Nursing and Midwifery, Faculty of Medicine and Health, The University of Sydney, Level 8, Susan Wakil Building, Sydney, NSW 2006 Australia; 2 Charles Perkins Centre, The University of Sydney, Sydney, Australia

**Keywords:** Youth, Adolescent, Participatory research, Advisory group, Consumer, Chronic disease prevention

## Abstract

Our comment discusses our experience establishing a youth advisory group focused on chronic disease prevention research. The comment highlights three key learnings: the need for researchers to adapt their working style, the importance of redefining the power dynamics, and disrupting traditional research structures to align with co-researcher engagement models.

## Abstract (plain language summary)

In this comment, we share our insights from working with a youth advisory group in adolescent health research. Our comment supports our research paper on co-designing an online health study, “Health4Me.” The youth advisors worked with us for a year, during which we also studied leadership and other outcomes.

Adolescent health is gaining worldwide attention. Health researchers see the value of working with young people on issues that affect them. To address this need, we formed the Health Advisory Panel for Youth at the University of Sydney. We motivated young people through building new skills and leadership training, rather than only focusing on improved health outcomes or health knowledge.

Here are our key lessons as researchers:

- Flexibility: We changed our approach to support online teamwork with young people, offered payment and worked around their schedules.
- Changing power dynamics: We empowered young people by engaging them in decisions and involved them in co-authoring papers and presentations.
- Challenging traditional structures: To support youth- as co-researchers, we used existing sources of funding and offered mentoring.

In conclusion, we highlight practical ways of working with a youth advisory group in health research. Our teamwork led to a youth digital health program, published essays, and future research ideas. It is important for researchers to actively involve and support young people in shaping research that affects them.

## Introduction

To complement the paper “Striking the right balance: co-designing the Health4Me healthy lifestyle digital health intervention with adolescents,” [1] this comment reflects on our experience as researchers in establishing and managing a youth advisory group. Our youth advisory group supported our research projects, including the Health4Me intervention [2] for 12 months in 2021/22. We have also evaluated the process of establishing and facilitating the youth advisory group, which has been published elsewhere and includes the effect of participation in the youth advisory group on adolescents’ leadership skills and perceptions related to chronic disease prevention research [3]. This comment extends and enriches our research findings by providing additional insights and reflections from our perspectives as researchers into the meaningful engagement of adolescents in health research that affects them.

Adolescent health and well-being are gaining attention globally with the “1.8 Billion Young People for Change Campaign” leading to the 2023 Global Forum for Adolescents [4]. Global organisations, including UNICEF, the World Health Organisation’s Partnership for Maternal, Newborn and Child Health, and the United Nations Convention on the Rights of the Child, recognise the need for younger generations to be involved in issues that impact them. In the face of intergenerational challenges such as the chronic disease epidemic, it is increasingly important for researchers to engage meaningfully with adolescents in research decision-making. In a prevention research context, adolescent engagement is a developing area of investigation with limited pragmatic evidence for researchers [5]. Our systematic review found few studies in the chronic disease prevention field that meaningfully engaged adolescents as co-researchers, and fewer still evaluated their experiences in such a role [6].

To address the limited evidence for meaningfully engaging adolescents in chronic disease prevention research, we established the Health Advisory Panel for Youth at the University of Sydney (HAPYUS, pronounced ‘Happy Us’) in October 2021. In brief, of the 16 members, the mean age was 16 years, 50% of members identified as female, 25% resided in rural and remote areas and approximately 40% spoke a language other than English at home. We sought to recruit a diverse group of young people as our research extends beyond healthcare services research and into areas such as the food environment and digital landscape that are relevant to adolescent health and well-being broadly. To engage adolescents as co-researchers, we focused on process motivators, such as learning new skills and gaining experience, over outcome motivators, such as improved health outcomes or health knowledge. This comment highlights three key learnings from our experience as researchers coordinating our youth advisory group. We also provide a summary of dilemmas experienced in relation to each key learning and strategies we employed to overcome these challenges (Table 1).

**Table 1:** Key learnings, dilemmas and strategies drawing on our experiences as researchers facilitating a youth advisory group

Key learnings	Experienced dilemmas	Strategies
Adapt working style	We faced the challenge of bringing 16 young people, who were previously unfamiliar with one another and cultivating a culture of mutual respect conducive to their roles as co-researchers. Acknowledging the casual nature of their involvement and their other commitments, we adapted our working style to ensure maximal engagement throughout the 12-month period	1. Employing a tiered approach to engagement, providing multiple avenues for participation during Zoom meetings, including verbal contributions, chat interactions, and the option to keep cameras on or off based on individual comfort levels
		2. Leveraging various features of Slack, such as group chats and private messaging by the moderator, to foster rapport among participants and stimulate engagement and collaboration
		3. Challenging traditional hierarchical communication structures (such as formal email communication) by fostering open dialogue and knowledge exchange on Slack, while maintaining a culture of respect and professionalism
		4. Striking a balance between the use of collaborative digital tools such Google Docs and Mural, ensuring the protection of sensitive research data through robust data privacy and security measures
Redefining power dynamics	We endeavoured to involve our youth advisors as co-researchers, extending the opportunity for co-authorship in scientific publications to those who met the authorship criteria. However, this presented a dilemma, as researchers, we have significant expertise in the scientific publication process, requiring us to mentor our youth advisors on navigating this complex process	1. Providing support and guidance to our youth advisors without imposing our own perspectives, ensuring their autonomy in shaping their contributions to the publications
		2. Assisting youth advisors in crafting statements to be included in published manuscripts (e.g. Boxes), thereby clearly delineating their contributions and interpretations of the findings
		3. Selecting journals with inclusive authorship policies that recognise the valuable input of young people or consumers, thereby fostering a supportive publishing environment
		4. Overcoming setbacks encountered with journals lacking such policies, which often categorised youth co-authors as research participants, necessitating additional ethics clearance and parental consent
		5. Advocating on behalf of our youth advisors to journals, emphasising their significant role and contributions to the research, thereby encouraging recognition and acceptance of their involvement
		6. Providing educational workshops or resources to youth advisors on the publication process to empower them with knowledge and skills
Disrupting traditional research structures	In our efforts to operate our youth advisory group effectively and equitably, we needed to secure adequate funding and research capacity, a challenge we addressed by leveraging an existing grant. However, this solution presented a dilemma: how could we ensure the sustainability of our 12-month youth advisory model to support our future research endeavours?	1. Conducting a formal evaluation to gather evidence on the benefits of youth involvement in research, ensuring safe and effective engagement—a crucial consideration for researchers seeking to engage young people meaningfully
		2. Promoting the involvement of youth advisors within our institution and beyond to ensure recognition of their valuable contributions, while advocating for the rights of young people to participate in matters that affect them directly
		3. Implementing succession planning to address the issue of youth advisors aging out of their roles, ensuring continuity and providing opportunities for new youth advisors to engage in research initiatives, thereby fostering a dynamic and inclusive research environment
		4. Creating a platform or network for former youth advisors to stay connected and continue contributing to research initiatives in advisory or mentorship roles, thereby harnessing their expertise and experience for the benefit of future projects

### Adapt working style

Firstly, we needed to adapt our working style to establish and foster our youth advisory group. The group operated mostly online as the adolescents were recruited from a wide geographical area in New South Wales, Australia. We provided monthly payments to youth advisors and allocated researcher time to support the group. Online meetings were scheduled outside school hours to accommodate the youth advisors’ schedules. We envisioned most of the research collaboration occurring during the scheduled monthly meetings. However, we realised that youth advisor attendance at these meetings was limited, with only half of the group attending on average. To address this, we adopted a tiered engagement approach, offering multiple avenues for youth advisors to contribute comfortably and safely. We also secured funding to host one full day in-person workshop with the program designed by the youth advisors. Costs covered travel for members residing in rural or regional areas to attend.

We used Slack and linked collaborative tools such as Google Docs and Mural. As well, we made the meeting recordings available on Slack. As younger millennials and Gen Z researchers, ourselves, we are accustomed to using such online collaborative tools. However, our research training and institutional culture favours more traditional forms of communication and collaboration, such as email and file sharing on internal servers, which we recognise is important for research data privacy and security. We learned to be flexible and agile in our approach, while considering data privacy and security and adjust to group engagement changes and we recognise this approach is different to how our traditional research team operates. We recommend that researchers allocate a lead moderator to facilitate safe collaboration online and effectively communicate with the youth advisors and use tools that support collaborative research, while also adhering to data security and privacy. A lead moderator can then allocate sufficient time to engage with group conversations, respond in a timely manner and help facilitate conversations and engagement, which helped reduce feelings of tokensim. Importantly, this allows youth advisors to build rapport with a key member of the research team. It is important to note, while we adapted our communication style, youth advisors also adapted their communication style as their initial approach was more aligned with how they might commonly communicate with teachers, whereas we were encouraging them to consider their role as co-researchers. We also recommend researchers evaluate their team’s current working style and adapt elements that are not inclusive or do not foster effective collaboration with external collaborators, such as youth advisors.

## Redefining power dynamics

Secondly, redefining the traditional power dynamic was crucial to engaging adolescents as co-researchers. Traditionally, adolescents are viewed as research participants rather than co-researchers. To shift this power gradient, we recruited them to include them in decision-making processes and value their lived experiences. To sustain a successful co-researcher partnership, researchers need to provide opportunities for adolescents to feel empowered within the scientific community, such as co-authorship on publications and reports and presentation opportunities. Our approach was guided by youth participatory action research (YPAR) principles [5], and we started by asking the youth advisors to identify their top issues related to chronic disease prevention. This task resulted in a published essay in a scientific journal [7] and presentations at national conferences (e.g., Australian Medical Association 2022 National conference) empowering youth advisors and demonstrating the value of their perspectives in the scientific community.

## Disrupting traditional research structures

Finally, challenging the traditional research structures, the principles of YPAR encourage adolescent involvement in the entire research cycle, from deciding on research questions to undertaking systematic research. However, this poses a dilemma when resources such as researcher time and funding that are required for authentic youth engagement in research question development are typically not readily available through conventional scientific systems. Traditionally, researchers have already decided what to research before engaging with consumers and then applied for funding through submitting a grant proposal for peer review, which can take a significant amount of time. In our case, we had received funding to develop a youth-centred digital health program, Health4Me [2]. We had already decided what to research, however, to overcome some of the obstacles, we included sufficient budget to support a youth advisory group that would support the Health4Me project and could be leveraged to support future research projects. Through this strategy, we not only contributed to the grant's objectives—co-designing a youth-centred digital health program—but also delved into issues important to our youth advisors resulting in a co-authored publication on their top issues of concern for chronic disease prevention [7]. Additionally, we generated evidence regarding the impact of youth advisory groups through a formal evaluation of the group, focusing on their leadership and research skills, which has been published elsewhere [3]. Research timeframes, such as funding calls and research projects, which can be sporadic or have unforeseen delays, make it challenging to align with a 12-month youth advisory group. YPAR remains a cyclical process of learning and action, but changes are necessary within scientific systems to accommodate adolescents as co-researchers and modernise the system itself.

## Conclusions

In conclusion, our comment sheds light on the practical considerations of establishing and facilitating a youth advisory group in the context of chronic disease prevention and digital health research. The three key learnings from our experience include the need for researchers to adapt their working style, redefine the traditional power dynamic between adolescents and researchers, and challenge conventional research structures to align with models of co-research, such as YPAR. Our successful collaboration between researchers and the youth advisors resulted in a youth designed digital health program, the publication of a scientific essay and future research ideas to explore. The global community recognises the importance of involving young people in issues that impact them. With growing attention on adolescent health and well-being, it is crucial for researchers to actively involve and support adolescents in making decisions about research that affects them.

## Data Availability

All data generated or analysed during this study are included in this published article and its supplementary information files.

## Abbreviations

HAPYUS: Health Advisory Panel for Youth at the University of Sydney
YPAR: Youth participatory action research
